# Bortezomib retreatment for relapsed and refractory multiple myeloma in real‐world clinical practice

**DOI:** 10.1002/hsr2.104

**Published:** 2018-12-07

**Authors:** Cyrille Hulin, Javier de la Rubia, Meletios A. Dimopoulos, Evangelos Terpos, Eirini Katodritou, Vania Hungria, Hadewijch De Samblanx, Anne‐Marie Stoppa, Jesper Aagesen, Deniz Sargin, Anastasia Sioni, Andrew Belch, Joris Diels, Robert A. Olie, Don Robinson, Anna Potamianou, Helgi van de Velde, Michel Delforge

**Affiliations:** ^1^ Service d'Hématologie Hôpital Haut‐Lévêque CHU Bordeaux France; ^2^ Department of Hematology, Hospital Dr Peset Universidad Católica de Valencia Valencia Spain; ^3^ Department of Clinical Therapeutics National and Kapodistrian University of Athens School of Medicine Athens Greece; ^4^ Department of Hematology, Theagenion Cancer Centre Thessaloniki Greece; ^5^ Santa Casa de São Paulo Medical School São Paulo Brazil; ^6^ Hematology, St Dimpna Hospital Geel Belgium; ^7^ Département D'Onco‐Hématologie Institut Paoli‐Calmettes Marseilles France; ^8^ Department of Medicine, Ryhov County Hospital Jönköping Sweden; ^9^ Division of Hematology, Department of Internal Medicine Istanbul University Istanbul Turkey; ^10^ Department of Oncology Cross Cancer Institute Edmonton AB Canada; ^11^ Janssen Research and Development, Division of Janssen Pharmaceutica NV Beerse Belgium; ^12^ Janssen‐Cilag AG Zug Switzerland; ^13^ Janssen Global Services Raritan NJ USA; ^14^ Janssen‐Cilag Pharmaceutical SACI Athens Greece; ^15^ Millennium Pharmaceuticals, Inc., Cambridge, MA, USA a wholly owned subsidiary of Takeda Pharmaceutical Company Limited; ^16^ Department of Hematology University Hospital Leuven Leuven Belgium

**Keywords:** multiple myeloma, real world, refractory, relapsed, retreatment

## Abstract

**Aims:**

Studies have shown that bortezomib retreatment is effective in relapsed/refractory multiple myeloma (MM). The observational, prospective electronic VELCADE^®^ OBservational Study (eVOBS) study assessed bortezomib‐based therapies for patients with MM in everyday practice. Here, we report on those patients receiving retreatment with bortezomib.

**Methods:**

Consenting adults scheduled to receive bortezomib for MM were enrolled at 162 sites across Europe, Canada, Brazil, Russia, and Turkey between 2006 and 2010. Retrospective data on prior therapies and prospective observational data after bortezomib initiation were captured electronically at baseline, after every bortezomib cycle, and every 12 weeks after discontinuation or progression. Investigator‐assessed responses and adverse events (AEs) were evaluated.

**Results:**

Ninety‐six of 873 patients enrolled to eVOBS received bortezomib as first retreatment for progressive disease during the prospective observation period. Median age was 62 years, 53% were male, and median number of prior therapies at retreatment was 4. Overall, 41% of patients initiated bortezomib retreatment in combination with dexamethasone, 16% in combination with lenalidomide, and 21% received monotherapy. Rate of partial response or better (≥PR) was 75% at initial bortezomib therapy, including 44% complete response (CR)/near CR (nCR); at retreatment, ≥PR rate was 46%, including 15% CR/nCR. Median progression‐free survival was 11.4 months (95% confidence interval [CI]: 9.1‐12.7) from start of initial bortezomib treatment and 6.4 months (95% CI: 4.4‐7.2) from start of retreatment. Median overall survival from start of retreatment was 17.6 months (95% CI: 14.4‐23.5). Of the 96 patients retreated with bortezomib, 77% reported an AE. Peripheral neuropathy during bortezomib retreatment occurred in 49% of patients, including 10% grade 3/4.

**Conclusion:**

These data suggest that retreatment with bortezomib is a feasible option for patients with relapsed/refractory MM.

## 1 INTRODUCTION

Multiple myeloma (MM) is an incurable disease with a high incidence rate in elderly people.^1^ The disease typically follows a relapsing course, with many patients requiring multiple lines of therapy.^2^ The choice of treatment for relapsed and/or refractory (RR) MM may be influenced by several factors, including patients' prior regimen(s), comorbidities, disease characteristics at relapse, prior treatment‐related toxicities, and duration of prior remission.^3^, ^4^, ^5^ One of the mainstays of treatment for RRMM is the proteasome inhibitor bortezomib, with numerous phase 2 and 3 studies clearly showing therapeutic effectiveness in this patient population.^6^, ^7^, ^8^, ^9^, ^10^, ^11^, ^12^, ^13^, ^14^, ^15^, ^16^, ^17^, ^18^, ^19^, ^20^ In Europe, bortezomib is currently approved for progressive MM in patients who have received ≥1 prior therapy (alone or in combination with pegylated liposomal doxorubicin or dexamethasone) and who have already undergone or are unsuitable for hematopoietic stem cell transplantation (HSCT). It is also approved as a treatment for patients with previously untreated MM who are ineligible for high‐dose chemotherapy with HSCT (in combination with melphalan and prednisone), or as induction treatment prior to high‐dose chemotherapy with HSCT (in combination with dexamethasone, or with dexamethasone and thalidomide).^21^ Bortezomib is a recommended treatment option for RRMM.^22^

For patients with MM who receive a finite course of bortezomib (ie, not receiving maintenance treatment), their disease may remain sensitive to bortezomib‐based therapy at relapse. Retreatment with bortezomib is, therefore, a viable option for patients with progressive disease (PD), either as a subsequent or later line of therapy after initial bortezomib treatment. A number of retrospective studies,^23^, ^24^, ^25^, ^26^, ^27^, ^28^, ^29^, ^30^, ^31^ prospective clinical trials,^32^, ^33^ and a recent meta‐analysis^34^ have demonstrated the viability of retreatment with bortezomib, all showing bortezomib‐based retreatment to be efficacious and tolerable.^35^ On the basis of the prospective clinical study by Petrucci et al,^32^ the indication for bortezomib in the US was expanded in late 2014 to include retreatment in patients who have previously responded to bortezomib and who have relapsed at least 6 months after completing prior bortezomib treatment.^36^

While the efficacy and safety of bortezomib‐based therapies for retreatment have been shown in the highly controlled clinical trial setting, these findings may not reflect those observed in routine medical practice, where the patient population can differ substantially from that selected by strict clinical trial entry criteria. To date, however, data on the use of bortezomib retreatment in the “real‐world” oncology practice setting are limited. To address this gap, we conducted a sub‐analysis of the prospective, international, non‐interventional, electronic VELCADE OBservational Study (eVOBS) that was designed to study the efficacy and safety of bortezomib‐based therapies for MM in real‐world medical practice.^37^ In our sub‐analysis, we examined the efficacy and safety of bortezomib‐based retreatment for relapsed MM during the monitoring period of eVOBS.

## 2 METHODS

### 2.1 Study design and patients

Electronic VELCADE OBservational Study was an open‐label, non‐interventional, observational study designed to collect prospective data from MM patients undergoing bortezomib‐based therapy within any of the locally approved indications in the real‐world oncology practice setting. Details of study design and conduct have been published recently.^37^ In brief, any adults initiating bortezomib treatment for MM in participating centers were eligible for inclusion and all those with at least a baseline assessment are reported here. All bortezomib doses and concomitant treatments (except investigational therapies) were permitted. Patients participating in any other investigational study, however, were ineligible.

Patients were enrolled between June 2006 and December 2010 at clinical practices in Belgium, Brazil, Canada, France, Greece, Russia, Spain, Sweden, and Turkey. The study was conducted in accordance with the Declaration of Helsinki, Good Clinical Practice, and applicable regulatory requirements, and was approved by an Independent Ethics Committee or Institutional Review Board in all participating countries. All patients provided written informed consent in accordance with local legislation.

### 2.2 Objectives and endpoints

The overall objective of eVOBS was to evaluate the clinical outcomes associated with bortezomib‐based therapies in real‐world medical practice.^37^ The objective of the present sub‐analysis was to evaluate the efficacy and safety of bortezomib‐based retreatment in patients with progressive MM following initial bortezomib‐based treatment during the prospective observational period of eVOBS. Data on the following endpoints were collected prospectively: response rates (complete response [CR], near‐CR [nCR], partial response [PR], minimal response [MR], stable disease [SD], and PD), time to response, treatment‐free interval (TFI), progression‐free survival (PFS), overall survival (OS), and safety.

### 2.3 Data collection and assessments

Patients' MM treatment histories during the year prior to starting bortezomib were recorded retrospectively. Additional information, including patient demographics and disease characteristics, scheduled bortezomib dose, concomitant medications, and laboratory parameters, were obtained at initiation of bortezomib treatment (baseline). Observational data were then collected prospectively over a 3‐year period following initiation of the first cycle of bortezomib. Data were captured electronically at baseline and after every bortezomib cycle, with the exception of serious adverse events (AEs), which were reported within 24 hours of the knowledge of the event. Any bortezomib dose adjustments or cycle delays were documented.

Each site used and recorded its own existing methods and criteria for response assessment. MM disease stage was assessed at the time of diagnosis using Durie‐Salmon or International Staging System criteria. Responses were assessed by investigators applying modified European Group for Blood and Marrow Transplant (EBMT),^38^ Southwest Oncology Group (SWOG),^39^ monoclonal protein (M‐protein) reduction,^40^ or other (not specified) criteria. Due to the non‐interventional nature of the study, no predefined response criteria were mandated. Critical definitions of response were not significantly different across all criteria used in this analysis whether EBMT, SWOG, M‐protein, or other criteria. AEs were graded per the National Cancer Institute Common Terminology Criteria for Adverse Events version 3.0. Upon discontinuation of bortezomib, data on subsequent therapies, survival, and disease progression were collected every 12 weeks, for up to 3 years, after bortezomib initiation.

### 2.4 Statistical analyses

All time‐to‐event endpoints were analyzed using Kaplan‐Meier and Cox proportional hazards regression analyses. Patients lost to follow‐up or who discontinued bortezomib treatment without a reason were censored in all time‐to‐event analyses. Kaplan‐Meier analyses were stratified according to baseline characteristics (including age, MM stage, line of therapy, creatinine clearance, and baseline albumin) and best response to bortezomib. The two‐sided log‐rank test was used to assess the significance of any differences between the stratified data; the conventional significance threshold of 0.05 was used across all analyses. As described in Terpos et al, missing data were not substituted nor imputed.^37^ Statistical analysis was performed using SAS version 9.2.

## 3 RESULTS

### 3.1 Patients

In total, 1573 patients who initiated bortezomib treatment for MM, at any of the 162 surveyed centers, were enrolled into the eVOBS registry. Due to concerns about data quality, 700 patients enrolled in Russia were excluded from the primary study analysis, leaving 873 evaluable patients. Demographics and baseline characteristics for the entire eVOBS population have been reported previously.^37^

Of the 873 patients with MM who received bortezomib‐based therapy during the 3‐year prospective observational phase, 96 (11%) underwent retreatment with bortezomib for PD during this period. The number of retreated patients enrolled by country was Belgium (*n* = 29), Brazil (*n* = 17), France (*n* = 11), Greece (*n* = 26), Spain (*n* = 4), Sweden (*n* = 4), and Turkey (*n* = 5). Although patients were also enrolled into the eVOBS study at clinics in Canada, none underwent bortezomib retreatment for PD during the prospective observational phase.

Demographics and baseline characteristics for the 96 retreated patients are summarized in Table 1. Median age was 62 years (range 34‐80), and 8 (7%) were aged ≥75 years. Approximately half (53%) of the patients were male and 19% and 58% had stage II or III disease at initial diagnosis, respectively.

**Table 1:** Patient demographics and baseline characteristics

Characteristic	Patients Receiving Bortezomib Retreatment *(N* = 96)
Median age, years (range)	62 (34‐80)
Male, *n* (%)	51 (53)
Disease stage at bortezomib initiation, *n* (%)^a^	
I	17 (18)
II	18 (19)
III	56 (58)
Unknown	5 (5)
Creatinine clearance at baseline, *n* (%)^b^	
<60 mL/min	29 (30)
≥60 mL/min	64 (67)
Median time since first treatment for MM, years (range)	2.0 (0‐12)
Median number of therapies prior to bortezomib retreatment, *n* (range)^c^	4 (2‐9)

Abbreviation: MM, multiple myeloma.

a Based on Durie‐Salmon or International Staging System criteria.

b Creatinine clearance data missing for three patients.

c Including initial bortezomib.

In general, patient characteristics were largely similar to the overall eVOBS population. Slightly more patients here had a later stage of disease at bortezomib initiation (overall population: 28% stage II, 48% stage III; retreated population: 19% stage II, 58% stage III), and the median number of prior therapies and time since diagnosis was higher. In the retreatment population, the median age (62 years) and proportion of males (53%) were both slightly lower than in the overall population (65 years and 58%, respectively).

### 3.2 Bortezomib retreatment

The majority of patients undergoing bortezomib retreatment received this therapy as their fourth or fifth line (Table 2). The most common bortezomib‐based regimens received at initial treatment and at retreatment were bortezomib plus dexamethasone (53% and 41%, respectively) and bortezomib monotherapy (each 21%) (Table 2). There was a notable increase in the use of bortezomib plus lenalidomide combination therapies between initial bortezomib treatment (2%) and retreatment (20%).

**Table 2:** Bortezomib treatment and retreatment history within eVOBS

Characteristic	Initial Bortezomib Treatment^a^ (*N* = 96)	Bortezomib Retreatment^a^ (*N* = 96)
Line of therapy, *n* (%)		
1^st^	1 (1)	0 (0)
2^nd^	43 (45)	0 (0)
3^rd^	32 (33)	13 (14)
4^th^	6 (6)	29 (30)
≥5^th^	4 (4)	44 (46)
Best supportive care	4 (4)	4 (4)
Unknown	6 (6)	6 (6)
Bortezomib regimen received, *n* (%)		
Bortezomib‐dexamethasone	51 (53)	39 (41)
Bortezomib monotherapy	20 (21)	20 (21)
Other bortezomib‐dexamethasone combinations	12 (13)	5 (5)
Bortezomib‐thalidomide (including dexamethasone combinations)	7 (7)	6 (6)
Bortezomib‐prednisone	3 (3)	4 (4)
Other bortezomib combinations	1 (1)	3 (3)
Bortezomib‐lenalidomide	2 (2)	19 (20)
Dose of bortezomib received at initiation, *n* (%)		
1.3 mg/m^2^	85 (89)	67 (70)
≤1.0 mg/m^2^	8 (8)	18 (19)
Other	3 (3)	11 (11)
Median number of bortezomib cycles received, *n* (range)	6 (1–24)	4 (1–12)
Reasons for bortezomib discontinuation, *n* (%)^b^		
Completed planned course of treatment	31 (32)	18 (19)
AE	19 (20)	18 (19)
Progressive disease	14 (15)	32 (34)
Not reported	8 (8)	2 (2)
In remission	6 (6)	4 (4)
Autologous stem cell transplantation	6 (6)	1 (1)
Death	0	8 (9)
Other^c^	12 (13)	13 (14)

Abbreviation: AE, adverse event.

a Percentages may not equal 100% due to rounding.

b 95 of 96 retreated patients had discontinued bortezomib‐based therapy at data cut‐off.

c Includes patient withdrawal, lost to follow‐up, and those with unreported reasons.

In total, 91 (95%) patients had received an alternative treatment for MM between initial bortezomib and bortezomib retreatment. Of these, 49 (51%) had one alternative treatment, 25 (26%) had two, 12 (13%) had three, and 5 (5%) had four intermediate treatments. The most common intermediate treatments were lenalidomide plus dexamethasone (*n* = 42, 44%), autologous stem cell transplantation (ASCT; *n* = 23, 24%), and thalidomide (monotherapy or in combination with dexamethasone; *n* = 21, 22%).

Compared with 85 (89%) who started initial bortezomib treatment at 1.3 mg/m^2^, 67 (70%) patients started bortezomib retreatment at 1.3 mg/m^2^ and 18 (19%) did so at a reduced dose (≤1.0 mg/m^2^) (Table 2). These were mostly, if not all, IV administrations, since the study was conducted during a time that IV infusion was the only approved mode of administration. Patients received a median (range) of 6 (1‐24) bortezomib cycles during initial bortezomib treatment, compared with 4 (1‐12) cycles at retreatment. In bortezomib‐retreated patients, discontinuations during initial bortezomib treatment and retreatment, respectively, were predominantly due to treatment completion (32% vs 19%), discontinuation due to AEs (20% vs 19%), and PD (15% vs 34%) (Table 2).

### 3.3 Best response to bortezomib retreatment

Criteria used for response assessment in the subset of bortezomib‐retreated patients were M‐protein reduction (*n* = 35, 36%), EBMT response criteria (*n* = 33, 34%), other (not specified) criteria (*n* = 24, 25%), and SWOG response criteria (*n* = 4, 4%). Overall, 75% of patients achieved a best response of ≥PR following initial bortezomib therapy, compared with 46% of patients who underwent bortezomib retreatment (Kaplan‐Meier estimates). This included 44% and 15% of patients with CR/nCR, respectively. Median time to ≥PR was 1.4 months (95% confidence interval [CI]: 1.2‐2.5) for initial bortezomib treatment and 1.7 months (95% CI: 1.3‐2.1) for retreatment.

**Figure 1:**
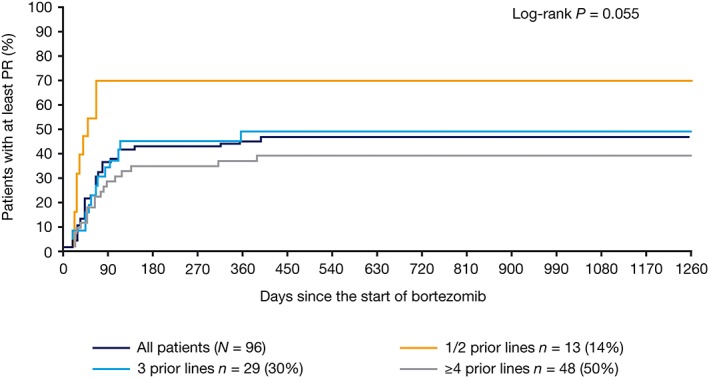
Kaplan‐Meier analysis of time to ≥PR from the start of bortezomib retreatment, stratified by number of prior lines of therapy. PR, partial response

We investigated factors that may influence response to retreatment. In total, 68% of patients who had achieved CR/nCR with initial bortezomib achieved ≥PR on retreatment; this figure was 39% in patients who had achieved PR and 20% in patients who had achieved ≤MR with initial bortezomib (*P* = 0.0022; two‐sided log‐rank test). In patients who had received one or two prior therapies, 70% achieved ≥PR at retreatment, compared with 48% for three prior therapies and 39% for four or more prior therapies (Figure 1; *P* = 0.055; two‐sided log‐rank test). There was no significant difference in the ≥PR rate to bortezomib retreatment in patients stratified by a TFI of <6 versus ≥6 months between end of previous treatment line and bortezomib initiation. Of the 96 patients receiving bortezomib retreatment, 12 received a second retreatment with bortezomib following PD, of whom one patient achieved CR and one achieved PR.

### 3.4 Survival outcomes

**Figure 2:**
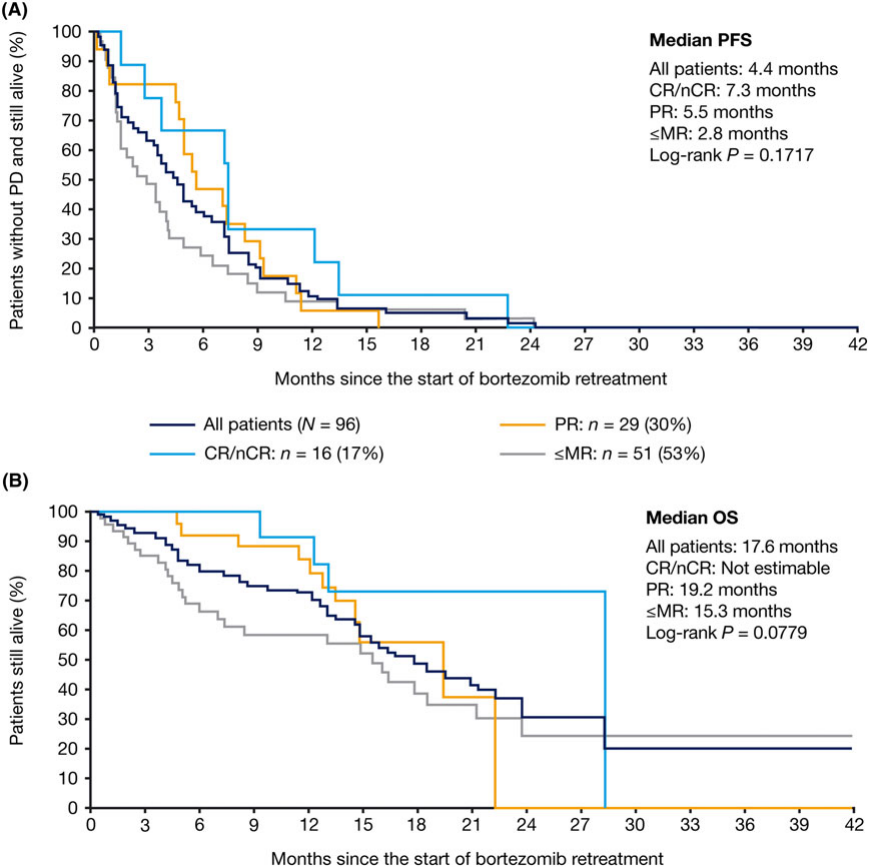
Kaplan‐Meier analysis of (A) progression‐free survival and (B) overall survival after bortezomib retreatment, stratified by best response to retreatment. CR, complete response; MR, minimal response; nCR, near complete response; OS, overall survival; PD, progressive disease; PFS, progression‐free survival; PR, partial response

In bortezomib‐retreated patients, median follow‐up from the start of initial bortezomib was 35 months (range 9‐60), and from the start of retreatment, 11 months (range 0‐30). All medians for time to event endpoints were generated using Kaplan‐Meier and Cox proportional hazards regression analyses. Median PFS in bortezomib‐retreated patients was 11.4 months (95% CI: 9.1‐12.7) from the start of initial bortezomib treatment and 6.4 months (95% CI: 4.4‐7.2) from the start of retreatment. Median OS was 41.8 months (95% CI: 33.7‐not estimable) from the start of initial bortezomib treatment and 17.6 months (95% CI: 14.4‐23.5) from the start of bortezomib retreatment. There was no statistically significant difference in PFS (*P* = 0.1717; two‐sided log‐rank test) and OS (*P* = 0.0779) from the start of bortezomib retreatment in patients who achieved CR/nCR versus PR versus ≤MR with bortezomib retreatment (Figure 2). Additionally, there was no statistically significant association between PFS (*P* = 0.9169; two‐sided log‐rank test) from the start of bortezomib retreatment and depth of response after initial bortezomib treatment.

**Figure 3:**
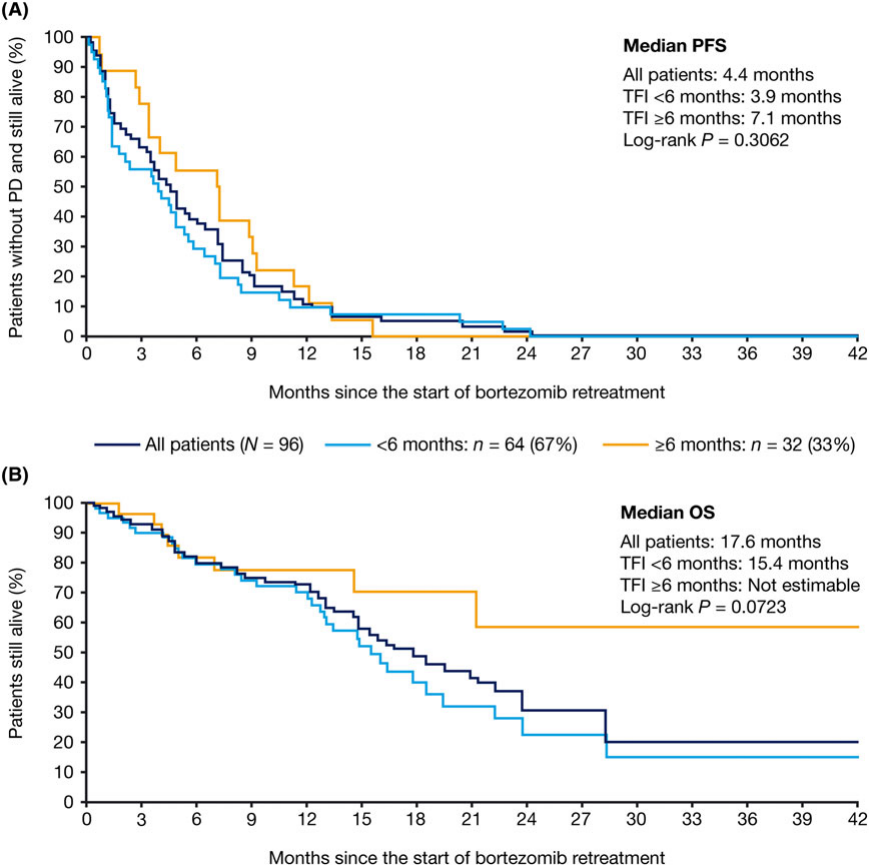
Kaplan‐Meier analysis of (A) progression‐free survival and (B) overall survival from the start of bortezomib retreatment, stratified by treatment‐free interval between initial bortezomib treatment and subsequent line of therapy. OS, overall survival; PD, progressive disease; PFS, progression‐free survival; TFI, treatment‐free interval

**Figure 4:**
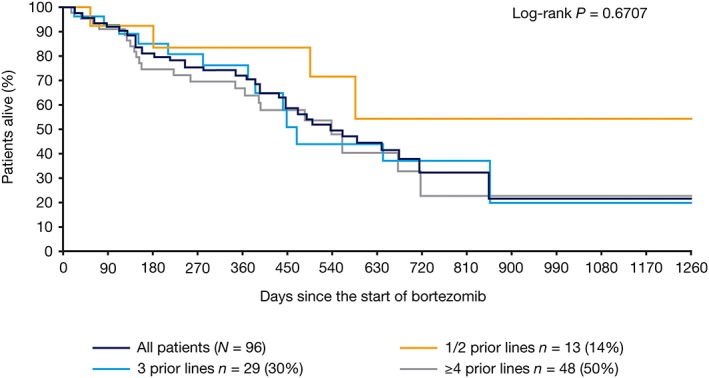
Kaplan‐Meier analysis of overall survival from the start of retreatment, stratified by number of prior lines of therapy

There was no statistically significant difference in OS (*P* = 0.0723; two‐sided log‐rank test), or PFS (*P* = 0.3062; two‐sided log‐rank test), from the start of retreatment in patients with a TFI of ≥6 versus <6 months between initial bortezomib and subsequent line of therapy (Figure 3). In addition, there was no significant difference in PFS (*P* = 0.9680, data not shown; two‐sided log‐rank test) or OS (*P* = 0.6707; Figure 4; two‐sided log‐rank test) according to the number of lines of therapy received prior to bortezomib retreatment.

### 3.5 Safety profile during retreatment

Of the 96 patients who underwent retreatment with bortezomib post‐PD, 74 (77%) reported AEs (Table 3). In total, 41 (43%) patients experienced grade ≥3 AEs, of which thrombocytopenia (5%) and anemia (4%) were most common. Serious adverse events (SAEs) were experienced by 39 (41%) patients, including pneumonia (6%), death (3%), disease progression (3%), and skeletal injury (3%). Eighteen (19%) patients discontinued bortezomib retreatment due to AEs, including neuropathy (*n* = 3), bone pain (*n* = 2), disease progression (*n* = 2), neutropenia (*n* = 2), and pneumonia (*n* = 2). Eight (8%) patients died during retreatment.

**Table 3:** AEs (≥5% of patients) reported with bortezomib retreatment

AE, *n* (%)	Any Grade (*N* = 96)	Grade ≥3 (*N* = 96)
Any AE	74 (77)	41 (43)
Neuropathy	24 (25)	3 (3)
Not otherwise specified	10 (10)	1 (1)
Aggravated	7 (7)	2 (2)
Peripheral sensory	7 (7)	0
Diarrhea	14 (15)	1 (1)
Fatigue	12 (13)	1 (1)
Pain	11 (11)	0
Upper respiratory infection	11 (11)	1 (1)
Nausea	10 (10)	1 (1)
Pneumonia	10 (10)	4 (4)
Thrombocytopenia	10 (10)	5 (5)
Asthenia	9 (9)	0
Back pain	8 (8)	1 (1)
Anemia	7 (7)	4 (4)
Neutropenia	7 (7)	3 (3)
Lower limb edema	7 (7)	0
Skeletal injury	7 (7)	4 (4)
Bone pain	6 (6)	2 (2)
Cough	6 (6)	0
Pyrexia	6 (6)	0
Appetite decreased	5 (5)	0
Headache	5 (5)	0
Infection	5 (5)	1

#### 3.5.1 Peripheral neuropathy

In the 96 bortezomib‐retreated patients, 66 (69%) had PN during initial bortezomib treatment (20% grade 1, 38% grade 2, 11% grade 3/4), while 47 (49%) had PN during bortezomib retreatment (18% grade 1, 21% grade 2, 10% grade 3/4) (Table 4). There was, therefore, no increase in PN incidence at retreatment versus initial treatment. Among the 30 patients without PN during initial bortezomib treatment, the rate of treatment‐emergent PN during bortezomib retreatment was 30%. Among the 66 patients with PN during initial bortezomib treatment, 38 (58%) also had PN during retreatment; of these, grade of PN was improved in 15 patients, worsened in 8 patients, and unchanged in 15 patients. The cumulative risk of developing PN plateaued after seven cycles of retreatment.

**Table 4:** Peripheral neuropathy during initial therapy and retreatment

Patients, *n* (%)		Worst PN during Retreatment					Totals
		No PN	Grade 1	Grade 2	Grade 3	Grade 4	
Worst PN during initial therapy	No PN	21 (22)	1 (1)	5 (5)	1 (1)	2 (2)	30 (31)
	Grade 1	11 (12)	4 (4)	3 (3)	1 (1)	0	66 (69)
	Grade 2	13 (14)	10 (10)	10 (10)	3 (3)	0	
	Grade 3	4 (4)	2 (2)	2 (2)	1 (1)	1 (1)	
	Grade 4	0	0	0	1 (1)	0	
Totals		49 (52)	47 (49)				96 (100)

Abbreviation: PN, peripheral neuropathy.

## 4 DISCUSSION

Results from this analysis of 96 patients retreated with bortezomib during the prospective observational phase of the eVOBS study suggest that bortezomib retreatment is feasible in patients with RRMM in routine medical practice with a safety profile consistent with previous studies of bortezomib.^21^ Over half of the retreated patients had advanced stage III MM at initial diagnosis, and the patient population, as a whole, was heavily pretreated before receiving bortezomib retreatment (median of four prior lines of therapy). Our findings compare favorably with those from the bortezomib pivotal phase 2 study, in which the ≥PR rate was 40% in 130 patients who had received a median of 2, rather than 4, prior lines of therapy,^32^ and are also in line with the results of other previous prospective and retrospective clinical studies.^23^, ^24^, ^25^, ^26^, ^27^, ^29^, ^33^, ^34^

The majority (77%) of patients included in this analysis were enrolled at clinics in countries within the European Union. Most of these patients received bortezomib‐dexamethasone or bortezomib monotherapy for initial bortezomib treatment, and bortezomib retreatment within eVOBS. These approaches are in line with the current European approval status of bortezomib^21^ and present treatment practices for RRMM in Europe.^22^

Of the 96 patients included in this study who were eventually retreated with bortezomib, 75% achieved ≥PR after their initial bortezomib treatment, including 44% CR/nCR. These rates are slightly higher than the 69% ≥PR rate and 37% CR/nCR rate observed in the overall eVOBS study population following initial bortezomib‐based treatment.^37^ Although the populations were generally similar, differences in patient and baseline disease characteristics between the overall study population and the retreated population may have contributed to these observed results.^37^ It is also likely that patients who had initially responded well to bortezomib were preferentially chosen for retreatment, resulting in a population with a higher initial response rate.

In our retreated cohort, 46% of patients achieved ≥PR with bortezomib retreatment, including 15% CR/nCR. The ≥PR rate with bortezomib retreatment reported here is comparable with overall response rates reported with bortezomib retreatment in previous prospective clinical trials,^32^, ^33^ a retrospective case series,^27^ and a meta‐analysis,^34^ but is slightly lower than the 60% reported by Ahn et al, in their retrospective study.^31^ In the latter study, however, patients had received a median of two prior therapies, and only those who had relapsed or progressed ≥6 months after the previous bortezomib therapy were included.^31^ Despite a higher response rate, median PFS was comparable, at 5.5 months (95% CI: 4.2‐6.8). Generally, though, due to differences in study design (eg, different criteria for response assessment and patient inclusion criteria/patient populations), inter‐study comparisons of response rates should be interpreted with caution.

The ≥PR rates observed with bortezomib retreatment in this study are encouraging, considering the advanced disease stage and heavily pretreated nature of the retreated population. This is consistent with previous studies demonstrating that bortezomib retreatment is feasible in later lines of therapy and can produce responses in a considerable proportion of patients.^24^, ^25^, ^27^, ^33^, ^41^ The observed decrease in ≥PR rate between initial bortezomib and bortezomib retreatment is consistent with the progressive nature of MM.^2^

Notably, 95% of patients retreated with bortezomib in eVOBS had received at least one alternative treatment for MM between initial bortezomib and bortezomib retreatment, which may have impacted on the observed ≥PR rate. Although infrequent in clinical practice, 12 patients in our population received a second retreatment with bortezomib after PD, with two patients going on to achieve ≥PR.

Consistent with previous prospective^32^ and retrospective studies,^23^, ^24^, ^28^, ^29^, ^31^ patient subgroup analyses showed that the response rate at retreatment was significantly higher in patients who achieved a deeper response with initial bortezomib. Yet, it is notable that 20% of patients with ≤MR to initial bortezomib achieved ≥PR following bortezomib retreatment, indicating that lack of a major response initially does not preclude a better response at a later stage. Many previous retreatment studies have included only those patients who achieved ≥PR upon their first therapeutic exposure to bortezomib. These observations suggest that at least a subset of the population with a best tumor response of MR on initial bortezomib treatment may benefit from subsequent retreatment. Results from two small retrospective bortezomib retreatment studies, conducted in the USA and the Republic of Korea, provide some supportive evidence for this hypothesis.^23^, ^31^ The clinical basis of this finding should be explored explicitly through larger prospective studies of bortezomib therapy in RRMM that are designed to include patients with any initial bortezomib response, including MR, to determine the most efficacious bortezomib retreatment combinations and their associated clonal dynamics.^42^, ^43^, ^44^ The CoMMpass trial is currently in progress and may suggest the optimal genotypic environment for bortezomib retreatment.^45^

In addition, there was a non‐statistically significant trend for a higher ≥PR rate in patients who had undergone fewer therapies prior to retreatment. While no significant difference in ≥PR rate was observed between patients who had a TFI ≥6 or <6 months in this study, higher overall response rates^24^ and longer OS^31^ in patients with a longer TFI have been reported previously.^23^

Limitations to the survey approach used for data collection in the eVOBS study include the variable criteria used for response assessment, which may have impacted the efficacy findings. However, this is the reality of clinical care across sites and countries, and although a substantial proportion of responses were assessed by M‐protein or non‐specified criteria, the overall eVOBS study population (*N* = 873) showed no substantial impact on survival distributions by best response (EBMT criteria vs other methods).^37^ Additionally, no formal sample size calculations were performed for the study population, which limited the ability to detect relevant changes pre‐ and post‐treatment.

Median PFS from the start of initial bortezomib (11.4 months) was longer than from the start of retreatment (6.4 months), in accordance with the disease course of an increasingly aggressive cancer in later lines of therapy.^2^ The median OS of 17.6 months from the start of bortezomib retreatment observed in this study is comparable with that reported in a recent meta‐analysis (16.6 months).^34^

Median number of cycles received at bortezomib retreatment was encouraging when compared with the number received at initial bortezomib treatment (4 vs 6 cycles, respectively). A lower proportion of patients received standard‐dose bortezomib (1.3 mg/m^2^) at initiation of retreatment compared with initial bortezomib treatment (70% vs 89%), which may have made a minor contribution to the observed lower response rates following retreatment. Although rates of discontinuation due to AEs were similar between bortezomib retreatment and initial bortezomib (19% vs 20%), a lower percentage of patients completed the planned course of treatment (retreatment: 19% vs initial: 32%), and discontinuations due to PD were higher (retreatment: 34% vs initial: 15%). These findings may reflect lower treatment tolerance, together with increased disease refractoriness, in this population of patients who had received multiple lines of therapy for MM prior to bortezomib retreatment.

The safety profile observed with bortezomib retreatment in this study is consistent with that known for bortezomib in RRMM,^12^, ^13^ and with prior clinical studies of bortezomib retreatment,^25^, ^28^, ^32^ and in a bortezomib retreatment meta‐analysis.^34^ The most common AEs reported with bortezomib retreatment in this study were hematologic‐, gastrointestinal‐, and neurologic‐related toxicities. Notably, there was no apparent increase in PN incidence at bortezomib retreatment versus initial bortezomib treatment. Although we did not document reversibility of PN, previous studies have shown that bortezomib‐induced PN is manageable and reversible in RRMM patients.^46^

In summary, the activity of bortezomib retreatment in this non‐interventional, observational study appears to reflect clinical trial experience to date. These data, obtained in a real‐world oncology practice setting, suggest that (1) retreatment with bortezomib is a feasible option for patients with RRMM, even among heavily pretreated patients, and (2) retreatment may produce better responses than originally achieved during the initial course of bortezomib treatment. Our results regarding survival among patients with deeper responses to bortezomib retreatment and/or a longer TFI following initial bortezomib treatment warrant further investigation.

If confirmed in future prospective studies, bortezomib retreatment for those patients responding below PR following initial bortezomib exposure would benefit from an additional treatment option. Furthermore, subcutaneous bortezomib administration is now available and has demonstrated improved tolerability compared with IV infusion.^47^ The role of second‐generation proteasome inhibitors in retreatment after an initial bortezomib course should also be investigated, as well as the potential clinical contribution of other combinatory agents in that setting, such as immunomodulatory therapies, histone deacetylase inhibitors, and chemotherapy.

## FUNDING INFORMATION

This analysis was supported by Janssen Research and Development.

## CONFLICTS OF INTEREST

None declared.

## DISCLOSURES

C. Hulin has received honoraria/consulting fees from Janssen, Celgene, Takeda, Amgen, and Novartis; J. de la Rubia has received personal fees from Amgen, Celgene and Janssen; M. Dimopoulos has received honoraria for participation in advisory boards with Janssen, Celgene, Takeda, Amgen, and Novartis; E. Terpos has received research funding from Amgen, Janssen, Takeda, Celgene/Genesis, honoraria from Amgen, BMS, Celgene/Genesis, Janssen, Novartis, Takeda, and GSK, is a steering committee member for Amgen and a data monitoring committee member for Celgene; E. Katodritou has received research support and personal fees from Janssen Cilag; V. Hungria has received personal fees for board membership and consultancy from Janssen‐Cilag, and payment for lectures including service on speakers' bureaus; A.‐M. Stoppa has received personal fees for consultancy and board membership from Janssen, Celgene, and Amgen; D. Sargin has received personal fees from Janssen; A. Belch has received personal fees from Janssen; J. Diels reports employment and shareholdings of Janssen, Johnson & Johnson; R.A. Olie reports employment and shareholdings of Janssen, Johnson & Johnson, and shareholdings of Amgen; D. Robinson Jr reports employment and shareholdings of Janssen, Johnson & Johnson; A. Potamianou reports employment and shareholdings of Janssen, Johnson & Johnson; H. van de Velde reports employment at Takeda Pharmaceuticals and stockholdings of Janssen, Johnson & Johnson; M. Delforge has received research grants from Janssen and Celgene, consulting fees/honoraria from Janssen, and personal fees from Janssen, Celgene and Amgen; H. De Samblanx, J. Aagesen, and A. Sioni have no conflicts to disclose. The financial relationships listed did not impact the conclusions of the study.

## AUTHOR CONTRIBUTIONS

Conceptualization: ET, JD, RO, AP

Formal Analysis: MeDi, ET, EK, AS, JD, RO, AP, HvdV, MiDe

Funding Acquisition: AP

Investigation: MeDi, ET, EK, VH, DS, AS, JD, RO

Resources: CH, JdlR, MeDi, HDS, AB

Writing—Original Draft Preparation: CH, JdlR, MeDi, ET, EK, VH, HDS, AMS, JA, DS, AS, AB, JD, RO, DR, AP, HvdV, MiDe

Writing—Review and Editing: CH, JdlR, MeDi, ET, EK, VH, HDS, AMS, JA, DS, AS, AB, JD, RO, DR, AP, HvdV, MiDe
